# Effectiveness and implementation of mPATH™-CRC: a mobile health system for colorectal cancer screening

**DOI:** 10.1186/s13063-023-07273-5

**Published:** 2023-04-14

**Authors:** Anna C. Snavely, Kristie Foley, Ajay Dharod, Mark Dignan, Holly Brower, Elena Wright, David P. Miller

**Affiliations:** 1 Department of Biostatistics and Data Science, Wake Forest University School of Medicine, Winston-Salem, NC USA; 2 Department of Implementation Science, Wake Forest University School of Medicine, Winston-Salem, NC USA; 3 Department of Internal Medicine, Wake Forest University School of Medicine, Winston-Salem, NC USA; 4 Department of Internal Medicine, College of Medicine, University of Kentucky, Lexington, KY USA; 5 Wake Forest University School of Business, Winston-Salem, NC USA

**Keywords:** Colorectal cancer, Screening, Implementation, Mobile health

## Abstract

**Background:**

Screening for colorectal cancer (CRC) is widely recommended but underused, even though CRC is the third most diagnosed cancer and the second leading cause of cancer death in the USA. The mPATH™ program is an iPad-based application designed to identify patients due for CRC screening, educate them on the commonly used screening tests, and help them select their best option, with the goal of increasing CRC screening rates.

**Methods:**

The mPATH™ program consists of questions asked of all adult patients at check-in (mPATH™-CheckIn), as well as a module specific for patients due for CRC screening (mPATH™-CRC). In this study, the mPATH™ program is evaluated through a Type III hybrid implementation-effectiveness design. Specifically, the study consists of three parts: (1) a cluster-randomized controlled trial of primary care clinics comparing a “high touch” evidence-based implementation strategy with a “low touch” implementation strategy; (2) a nested pragmatic study evaluating the effectiveness of mPATH-CRC™ on completion of CRC screening; and (3) a mixed-methods study evaluating factors that facilitate or impede the maintenance of interventions like mPATH-CRC™. The primary objective is to compare the proportion of patients aged 50–74 who are eligible for CRC screening who complete mPATH™-CRC in the 6th month following implementation between the “high touch” and “low touch” implementation strategies. Effectiveness of mPATH™-CRC is evaluated by comparing the proportion who complete CRC screening within 16 weeks of their visit to the clinic between a pre-implementation cohort (8 months before implementation) and a post-implementation cohort (8 months after implementation).

**Discussion:**

This study will provide data on both the implementation of the mPATH™ program and its effectiveness in improving screening rates for CRC. In addition, this work has the potential to have an even broader impact by identifying strategies to support the sustained use of other similar technology-based primary care interventions.

**Trial registration:**

ClinicalTrials.gov NCT03843957. Registered on 18 February 2019.

## Introduction

### Background and rationale {6a}

Screening for colorectal cancer (CRC) is widely recommended but underused, even though CRC is the third most diagnosed cancer and the second leading cause of cancer death in the USA [1]. Several professional groups, including the United States Preventive Services Task Force (USPSTF), have recommended regular screening for CRC beginning at age 50 [2–5], as CRC screening tests reduce both CRC mortality and incidence while being cost-effective [6–9]. In 2018, the American Cancer Society lowered the recommended age to initiate screening to age 45, and the USPSTF followed suit in 2021 based on a moderate net benefit of screening in this age group [10]. However, 32% of Americans are still unscreened despite recommendations [11].

There are many known barriers to CRC screening. These include patient factors such as lack of knowledge, low self-efficacy, fears, and negative attitudes [12–18], as well as provider or system factors such as lack of time, failure to offer screening options, and lack of patient support [19–23]. In addition, CRC screening rates are lower among underserved populations [24, 25], including in patients with less education, limited income, or rural residence [26–30]. Limited health literacy, which affects over 33% of American adults, is another challenge [31–35]. Therefore, meaningful increases in CRC screening will require an easily implemented intervention that addresses patient, provider, and system barriers while also being accessible to low-income and low-literacy individuals [36, 37]. The mPATH™ (mobile PAtient Technology for Health) program was designed to address these needs. mPATH™ is an iPad-based application, designed using plain language and lay terms, which patients use during routine health care visits [38]. Because most Americans over age 50 have seen a doctor within the past year, including 75% of adults with less than a high school education, interventions in medical practices have a potentially broad reach [39]. In addition, the mPATH™-CRC program (a module designed specifically to address CRC screening) uses a validated decision aid to inform patients of the commonly used screening tests and help them select their best option [40]. This is important because while several tests exist for CRC screening, most clinicians encourage colonoscopy, the most invasive and costly option [23, 41]. However, CRC screening rates are higher when patients are given a choice of testing options, so there is a critical need for interventions to help patients decide which test they prefer [24, 42]. In a previous randomized, controlled efficacy trial of 450 patients, mPATH™-CRC doubled CRC screening rates (30% vs 15%, *p*=0.0001) [43]. This efficacy trial demonstrated the potential for mPATH™-CRC to improve screening rates, but the implementation was led by a research team, thereby restricting its potential for scale-up in a real-world context. This major gap is now addressed in the current Type III hybrid implementation-effectiveness trial.

In September 2016, the Cancer Moonshot Blue Ribbon Panel’s report highlighted excessive mortality from CRC, the problems of health disparities, the need for interventions that inform patients of CRC screening tests, and the need for research to rapidly translate evidence-based CRC screening interventions into practice [44]. The study reported here directly addresses all four of these Cancer Moonshot priorities by examining the implementation of an innovative mobile health (mHealth) CRC screening intervention that is accessible by members of health-disparate populations. Translating mPATH™-CRC into widespread use could increase receipt of CRC screening in the USA to over 70%, the target set by Healthy People 2020 [45]. However, the optimal strategies for implementing mHealth interventions in clinical care remain unknown. Three recently published reviews identified over 500 articles and conference proceedings on mHealth studies, none of which prospectively examined competing implementation strategies [46–48]. Accordingly, this study has the potential to impact public health in two key ways: implementing mPATH™-CRC in community practices to decrease CRC mortality, and determining the best strategies for implementing and sustaining the growing number of other mHealth interventions that currently lack an evidence base for implementation.

### Objectives {7}

The overall goal of this study is to evaluate both the implementation and effectiveness of the mPATH™ program, which consists of two modules: mPATH™-CheckIn that asks routine questions of all adult patients presenting for a primary care visit, and mPATH™-CRC that is specific for patients due for CRC screening. To accomplish the overall goal, this study consists of three parts: (1) a cluster-randomized controlled trial of primary care clinics comparing a “high touch” evidence-based mHealth implementation strategy with a “low touch” implementation strategy; (2) a nested pragmatic study evaluating the effectiveness of mPATH™-CRC on completion of CRC screening; and (3) a mixed-methods study evaluating factors that facilitate or impede the maintenance of mHealth interventions like mPATH™-CRC. This study utilizes a Type III hybrid implementation-effectiveness design, meaning the primary objective is related to implementation, and a key secondary objective addresses effectiveness. The specific objectives are:

#### Primary objective

In a cluster-randomized controlled trial of 22 primary care clinics, compare the proportion of patients aged 50–74 who are eligible for CRC screening who complete mPATH™-CRC in the 6th month following implementation between the “high touch” and “low touch” implementation strategies.

#### Secondary objectives

- Compare implementation outcomes (Reach, Adoption, Acceptability, Appropriateness, Feasibility, Fidelity, and Maintenance) for mPATH™-CRC and mPATH™-CheckIn between the “high touch” and “low touch” implementation strategies in a cluster-randomized controlled trial of primary care clinics.
- Evaluate the effectiveness of mPATH™-CRC in a nested pragmatic study by comparing the proportion who complete CRC screening within 16 weeks of their visit to the clinic between a pre-implementation cohort (8 months before implementation) and a post-implementation cohort (8 months after implementation). The proportion who have a CRC screening test ordered will also be captured. Pre- vs post-implementation changes will be compared between the “high touch” and “low touch” implementation strategies and between dose levels defined based on mPATH™-CRC usage.
- Determine the factors that facilitate or impede the maintenance of mHealth interventions like mPATH™-CRC through in-depth qualitative interviews with clinic staff, providers, and administrators.

#### Exploratory objectives

- Compare implementation cost for the mPATH™ program between the “high touch” and “low touch” implementation strategies.
- Determine the factors that facilitate or impede the maintenance of mHealth interventions like mPATH™-CRC through clinic personnel surveys.

### Trial design {8}

This is a Type III hybrid implementation-effectiveness trial with the following parts:

1. *A cluster-randomized controlled trial.* In this implementation trial, 22 primary care clinics are randomized 1:1 to either a “high touch” implementation strategy or a “low touch” implementation strategy. Data are collected from clinics on implementation outcomes (Reach, Adoption, Fidelity, Maintenance, and Cost) over the 12 months following implementation. Clinic personnel surveys are administered (pre-implementation and 6- and 12-month post-implementation) to further evaluate implementation (Acceptability, Appropriateness, and Feasibility) from the staff perspective and to assess organizational characteristics, individual characteristics, and experience with the mPATH™ program.
2. *A nested pragmatic study.* To determine the effectiveness of mPATH™-CRC, a pragmatic trial is nested within the implementation study. Data from the electronic health record (EHR) at each study clinic are used to identify unscreened individuals aged 50–74 who completed a clinic visit in a pre-implementation cohort (months 12–4 before implementation) and a post-implementation cohort (months 1–8 after implementation). The EHR is then queried to determine whether these individuals complete CRC screening within 16 weeks of their visit.
3. *A mixed-methods study.* In-depth interviews (12-month post implementation or at drop-out) with clinic staff, providers, and administrators and clinic personnel surveys are used to examine the facilitators and barriers to maintenance (sustained use of mPATH-CRC™ over time).

A pilot phase preceded the launch of the cluster-randomized trial. During this phase, two primary care clinics were included to pilot test training materials and support for the “high touch” strategy, surveys, and the mPATH™ program.

## Methods: participants, interventions, and outcomes

### Study setting {9}

This study is being conducted in community-based primary care practices in North Carolina (affiliated with Atrium Health Wake Forest Baptist). Practices are chosen to reflect diversity in geography (urban, suburban, and rural), clinic structure, and clinic populations served.

### Eligibility criteria {10}

This study includes three distinct participant groups:

1. *Healthcare providers and staff at primary care practices.* All clinic personnel (e.g., administrators, nurses, providers [physicians, nurse practitioners, and physician's assistants]) who are involved with the implementation of the mPATH™ program receive surveys. In each clinic selected for qualitative interviews, semi-structured interviews are also conducted with four clinic members: the clinic champion, one clinician, one front desk team member, and one medical assistant/nursing team member.
2. *Patients aged 18 and older seen in the participating study sites.* The following eligibility criteria apply for patients to be a part of the study population to evaluate the implementation of mPATH™-CheckIn at participating study clinics:
  - Age ≥18
  - Spanish or English-speaking
3. *Patients aged 50–74 seen in the participating study sites who are eligible for CRC screening.* The following eligibility criteria apply for patients to be a part of the study population to evaluate the implementation and effectiveness of mPATH™-CRC at participating study clinics. The age inclusion criteria reflect the USPSTF guidelines that were in effect when the study commenced.
  - Age 50–74
  - Spanish or English-speaking
  - Due for routine CRC screening, defined as:
    No colonoscopy within the prior 10 years
    No flexible sigmoidoscopy within the prior 5 years
    No computed tomography (CT) colonography within the prior 5 years
    No fecal deoxyribonucleic acid (DNA) testing within the prior 3 years
    No fecal blood testing (guaiac-based test with home kit or fecal immunochemical test) within the prior 12 months

### Who will take informed consent? {26a}

Patients are participating in a pragmatic trial in which mPATH™ is delivered as part of usual care and patient data collection for clinical outcomes occurs by retrospective electronic chart review. All patients receive current guideline-recommended care. Therefore, this trial is being conducted under a waiver of patient informed consent. Clinic staff and providers participate in surveys and potentially interviews. No sensitive information is collected from clinic staff or providers. Therefore, this trial is being conducted under a waiver of signed consent. All participating staff and providers receive a study information sheet explaining the purpose of the study, the nature of the data to be collected, and the voluntary nature of their participation.

### Additional consent provisions for collection and use of participant data and biological specimens {26b}

There are no additional provisions for the collection and use of participant data beyond those described in the previous section. This trial does not involve collecting biological specimens for storage.

## Interventions

### Explanation for the choice of comparators {6b}

As described in the “Background and rationale {6a}” section, optimal strategies for implementing mHealth interventions in clinical care remain unknown. Therefore, this study aims to compare two different implementation strategies: a “high touch” evidence-based mHealth implementation strategy and a “low touch” implementation strategy.

### Intervention description {11a}

mPATH™-CRC is a self-administered iPad program designed to be used in primary care clinics to improve CRC screening rates. Based on feedback provided by clinic staff through focus groups and semi-structured interviews [49], the original mPATH™-CRC program was adapted to meet check-in needs of the clinics. Therefore, in the current trial, the mPATH™ program is implemented in two parts: mPATH™-CheckIn and mPATH™-CRC. The mPATH™ program runs outside the EHR but directly receives and sends data to the EHR via application programming interfaces (APIs). The program was developed within the context of the Epic EHR, a commonly used EHR in the USA [50], and the platform used by all Atrium Health Wake Forest Baptist-affiliated practices. All clinics can access their data related to mPATH™ implementation and generate customized reports via a secure web interface.

#### mPATH™-CheckIn

Clinic staff hand the mPATH™-CheckIn program to all adult patients upon check-in for an appointment with a provider so they may use it immediately before their medical visit. mPATH™-CheckIn includes standard screening questions related to falls, safety and depression [51]. One question is displayed per screen, with large intuitive response buttons. For patients who are 50–75, an API reads in patients’ CRC screening history from the EHR. If no recent screening for CRC is found, the mPATH™-CheckIn program determines and notifies patients of their screening status (due or current) by asking patients three items about their screening history. If patients identify a specific risk factor (personal history of CRC, personal history of colorectal polyps, family history of CRC, or visible blood in stool), they are told they are due for screening and should discuss screening with their provider, because screening guidelines differ for these high-risk individuals [2, 4] and the mPATH™-CRC module would not be appropriate. mPATH™–CheckIn also checks its usage database on startup and only ascertains patients’ screening status if the patient has not completed the CRC questions in the prior 6 months, an approach validated in a prior study of a substance abuse screening system in primary care practices [52]. After completing questions in the mPATH™-CheckIn module, the iPad is returned to the front desk. Nursing staff are then able to import patient responses into the EHR upon taking them to an exam room. If the patient is due for CRC screening, a pop-up alert notifies the nursing staff who then launch the mPATH™-CRC module.

#### mPATH™-CRC

The mPATH™-CRC module displays a brief CRC screening decision aid video (both English and Spanish versions are approximately 5 min) that includes graphics, animations, and video testimonials from patients and physicians, consistent with Social Cognitive Theory [53, 54]. Both videos were developed based on qualitative testing with patients. The videos inform patients of the benefits and risks of screening (including costs and complications) and review the two most commonly used tests in the USA (colonoscopy and fecal immunochemical testing). After the video, mPATH™-CRC lets patients “self-order” screening tests [5, 40]. Self-ordered tests are automatically routed to the patient’s primary care provider for approval via the EHR, allowing providers to overrule an order if it is felt to be inappropriate. When patients are done using mPATH™-CRC, a clinic staff member collects the iPad. mPATH™-CRC automatically sends data wirelessly to a central server and deletes any onboard device data once transmission confirmation is received, thereby preventing any data loss.

#### Framework for implementation

**Fig. 1:**
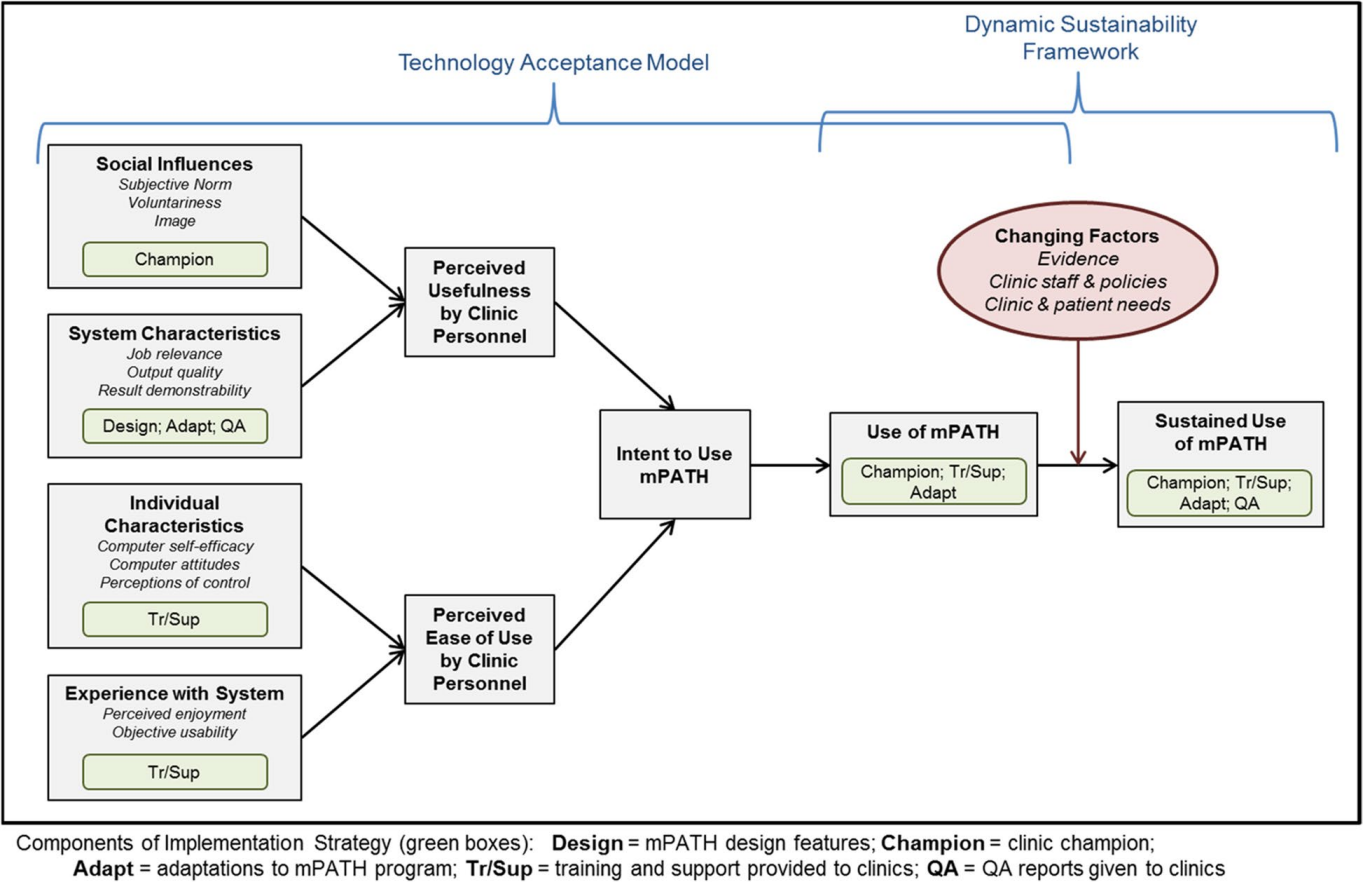
mPATH™ implementation theoretical model and strategies

Implementation of the mPATH™ program is based on the Technology Acceptance Model (TAM) [55–57] to guide initial implementation and the Dynamic Sustainability Framework [58] to guide maintenance, or continued use over time. The TAM posits that implementation of a new technology is determined by social influences and characteristics of the technology (which determine its *perceived usefulness*) and employees’ characteristics and experiences (which determine its *perceived ease of use*). After implementation, the Dynamic Sustainability Framework incorporates *adaptability* of mPATH™ as a means to promote maintenance. The “high touch” implementation strategy leverages evidence-based implementation strategies in conjunction with these two models (Fig. 1). Each clinic has a *clinic champion* who guides implementation and contributes to the Social Influence construct of the TAM. The research team provides facilitation through a pre-implementation meeting with the clinic champion, an on-site training session, “at-elbow” on-site support on the day of launching mPATH™, and regular support and follow-up phone conferences with the champion. During the follow-up phone conferences, topics known to promote implementation and sustainability are discussed, including reviewing quality assurance (QA) reports, identifying performance gaps, and brainstorming barriers [59–63]. Clinics can also use a reporting function in mPATH™ to generate custom reports of data collected and receive “real time” feedback. We expect that these features, and adaptations to mPATH™-CRC to meet check-in needs, will increase the perceived usefulness of the program. Training and support through a hands-on kick-off session, follow-up technical assistance, and repeated trainings as needed will increase staff self-efficacy, perceptions of control, and usability [60, 64]. Altogether, these multiple activities will increase intent to use mPATH™, use of mPATH™, and sustained use of mPATH™.

#### “High touch” strategy

Clinics randomized to the “high touch” strategy have pre-implementation activities, training, and ongoing support (Table 1).

*Pre-implementation activities*

At each site, a clinic champion is identified who will guide the local implementation effort. Champions typically arise through self or peer-nomination [65]. This person typically plays a central role in implementing new systems and has a demonstrated ability to communicate effectively, navigate the organizational environment, promote a project, and work well with others [65, 66]. The champion is identified by asking clinic administrators, lead nurses, and lead physicians who meet these qualities and would be willing to serve in this role. Each champion then helps the team identify a nursing super-user and a front desk super-user. Lastly, each clinic identifies an alternate champion to assist with implementation and provide continuity if the champion is unavailable due to vacation or illness. The alternate champion may be one of the super-users.

*Kick-off training session*

Two members of the study team visit each practice to conduct a training session for clinic staff identified by the local clinic champion. This training includes hands-on practice with mPATH™-CheckIn and mPATH™-CRC to increase staff’s comfort with the program, increase their self-efficacy, and demonstrate its usability. A separate brief training session is held for the clinic providers. The provider training includes an overview of the mPATH™ program and its functionality. Because providers will not directly interact with the mPATH™ program, the provider training does not include hands-on practice scenarios.

*At-elbow support*

The study team provides at-elbow technical support the day the clinic launches mPATH™ in their practice.

*On-going support*

The study team conducts a conference call with the clinic champion and alternate 2–3 business days after the launch date, and then at weeks 2, 4, 8, 16, and 24 post-launch to discuss the clinic’s experiences with mPATH™, review QA reports generated from mPATH™ data (frequency of use, patient demographics), explore the need for adaptations, and provide support. If program usage is below goal levels, barriers are reviewed with the clinic champion and additional on-site training is offered. Monthly “shout-out” memos are also provided to highlight clinic and staff performance. Clinics may request technical support and additional training at any time.

#### “Low touch” strategy

Clinics randomized to the low touch implementation strategy receive: (1) the initial kick-off on-site training session and implementation materials (identical to the “high touch” group), (2) access to phone/email technical support as requested, and (3) access to QA data (Table 1). Study staff will not proactively reach out to “low touch” clinics after the kick-off training session.

**Table 1:**
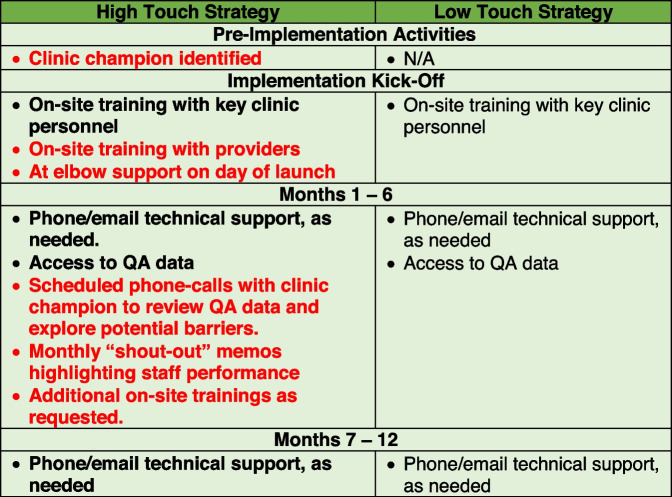
Comparison of implementation strategies

Red text indicates items unique to high touch strategy

### Criteria for discontinuing or modifying allocated interventions {11b}

The research team provides a suggested workflow for mPATH™ implementation at clinics. In this workflow, mPATH™-CheckIn is handed out by front desk staff and completed by patients in the waiting room. The nursing staff then transmit the data to the EHR, review their responses, and launch the mPATH™-CRC module for patients who are due for CRC screening. Patients then use mPATH™-CRC in the exam room while they wait for their doctor. However, clinics have the ability to modify this workflow as needed for their particular setting. For “high touch” clinics, the research team helps brainstorm implementation modifications to address any barriers identified during follow-up phone calls. Clinics can discontinue the use of mPATH™ entirely at their site, or individual patients can opt not to use the program.

### Strategies to improve adherence to interventions {11c}

To help improve the use of the mPATH™ program, phone and technical support is provided as needed to all clinics participating in the study. In addition, all clinics have access to QA data after implementation. “High touch” clinics have additional support as outlined in the “Intervention description {11a}” section, including a discussion of mPATH™ usage data during follow-up phone calls.

### Relevant concomitant care permitted or prohibited during the trial {11d}

This trial is being conducted within the context of routine care delivery in primary care settings. Usual care will continue throughout the trial, with nothing prohibited.

### Provisions for post-trial care {30}

Because this trial occurs in the context of routine care delivery, there are no special post-trial provisions.

### Outcomes {12}

#### Implementation outcomes

Implementation outcomes for this study were guided by the RE-AIM framework [67].

The primary outcome for the trial is Reach of mPATH™-CRC, defined as the proportion of patients aged 50–74 who are eligible for CRC screening who complete mPATH™-CRC in the 6th month following implementation. Patients who use mPATH™-CheckIn and are told they are due for CRC screening but advised to discuss screening with their doctor due to detected risk factors are also counted as having used mPATH™-CRC.

Secondary implementation outcomes:

- mPATH™-CRC Reach (by month): The proportion of patients aged 50–74 who are eligible for CRC screening who complete mPATH™-CRC or have risk factors identified by mPATH™-CheckIn in months 1–5 following implementation
- mPATH™-CRC Reach (by socioeconomic strata): The proportion of patients aged 50–74 who are eligible for CRC screening who complete mPATH™-CRC or have risk factors identified by mPATH™-CheckIn in months 1–6 by socioeconomic strata; this outcome will also be calculated among CheckIn users only
- mPATH™-CRC Adoption: The mean usage of mPATH™-CRC among staff and providers over the first 6 months following implementation; usage is calculated for each staff/provider as the proportion of times mPATH™-CRC is completed out of the total times mPATH™-CRC should have been launched.
- mPATH™-CRC Acceptability, Appropriateness, and Feasibility: Mean Acceptability of Intervention Measure (AIM), Intervention Appropriateness Measure (IAM), and Feasibility of Intervention Measure (FIM) scores [68] for mPATH™-CRC as assessed on the 6-month clinic personnel survey
- mPATH™-CRC Implementation Fidelity: The proportion of patients who use mPATH™-CRC and request a CRC screening test who have a test ordered or have the order dismissed (i.e., “self-order” feature is used as designed) in months 1–6
- mPATH™-CRC Maintenance: The proportion of patients aged 50–74 who are eligible for CRC screening who complete mPATH™-CRC or have risk factors identified by mPATH™-CheckIn in months 7–12
- mPATH™-CheckIn Reach: The proportion of patients aged 18 or older who complete mPATH™-CheckIn in months 1–6; this outcome will be calculated overall and within socioeconomic strata
- mPATH™-CheckIn Adoption: The mean usage of mPATH™-CheckIn among staff and providers over the first 6 months following implementation; usage is calculated for front desk staff as the proportion of times mPATH™-CheckIn is completed out of the total times mPATH™-CheckIn should have been handed out; usage is calculated for nurses/providers as the proportion of times mPATH™-CheckIn is completed and data is transmitted to the EHR out of the total times mPATH™-CheckIn should have been handed out
- mPATH™-CheckIn Acceptability, Appropriateness, and Feasibility: Mean AIM, IAM, and FIM scores for mPATH™-CheckIn as assessed on the 6-month clinic personnel survey
- mPATH™-CRC Maintenance: The proportion of patients aged 18 or older who complete mPATH™-CheckIn in months 7–12

Exploratory implementation outcome:

- mPATH™ Implementation Cost: Cost to implement and maintain usage of the mPATH™ program from the perspective of a healthcare system considering implementation, including hardware (i.e., iPads, cases, charging cabinets), cloud data storage fees, training, and technical support. Clinic staff training costs will be computed using training time and national average wage values for nursing and other staff from the Bureau of Labor Statistics. Technical support costs will include travel and support staff time costs associated with the initial training, and any follow-up trainings. Ongoing costs to maintain usage of mPATH™ include costs to train new employees or replace hardware and technical support costs related to follow-up training, troubleshooting, and adapting the program as needed.

#### Effectiveness outcomes

Effectiveness of mPATH™-CRC is evaluated based on completion of CRC screening as determined through the nested pragmatic study.

A key secondary outcome is mPATH™-CRC Effectiveness, which is defined as the proportion of patients aged 50–74 who are eligible for CRC screening who complete CRC screening within 16 weeks of their visit to the clinic. Effectiveness is determined by comparing the proportion who complete screening in a pre-implementation cohort (months 12–4 before implementation) to a post-implementation cohort (months 1–8 after implementation).

An additional secondary outcome is having a CRC screening test ordered. The outcome is defined as the proportion of patients aged 50–74 who are eligible for CRC screening who have a CRC screening test ordered (colonoscopy, flexible sigmoidoscopy, fecal testing for blood, or fecal DNA testing) within 16 weeks of their visit to the clinic. This outcome will also be compared between the pre- and post-implementation cohorts.

#### Mixed methods outcomes

As a secondary outcome, facilitators and barriers to maintenance (sustained use of mPATH™-CRC over time) will be identified through semi-structured interviews. Interviews will explore how mPATH™-CRC was incorporated in the clinic’s work flow and factors that affected maintenance such as intervention adaptations, organizational characteristics, and the champion's role. Interviews will be conducted with four members of each selected clinic: the clinic champion, one clinician, one front desk team member, and one medical assistant/nursing team member. To supplement the qualitative data collection, quantitative data on facilitators and barriers will be collected through the clinic personnel interviews as an exploratory outcome.

### Participant timeline {13}

**Fig. 2:**
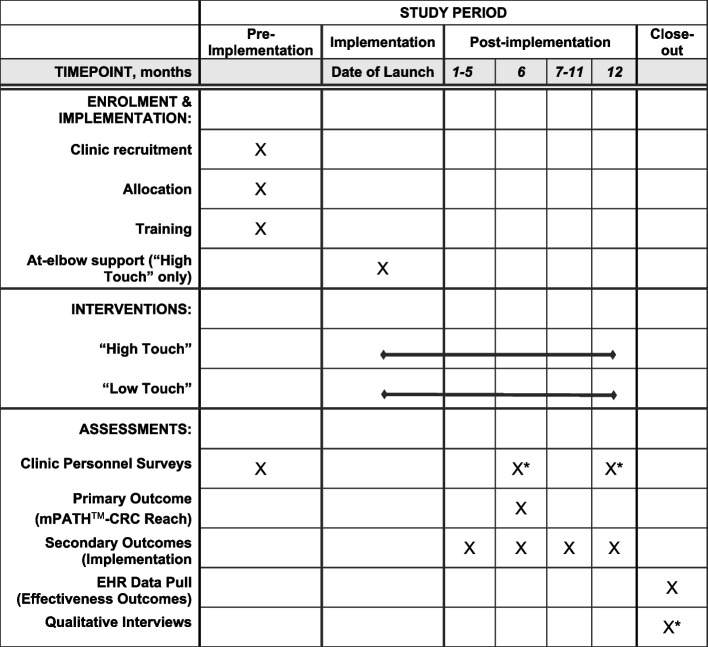
SPIRIT diagram. *Or as soon as possible after clinic drop-out

The timeline is illustrated in Fig. 2 below.

### Sample size {14}

#### Primary outcome (mPATH™-CRC Reach)

The primary objective is to assess differences in mPATH™-CRC Reach (proportion of participants who complete the mPATH™-CRC program or have risk factors identified by mPATH™-CheckIn) in the 6th month following implementation between the “high touch” and “low touch” strategies. Using formulae found in Hemming et al. [69], Table 2 shows the number of clinics necessary to detect absolute differences in Reach of 15% and 20% with 80% and 90% power, for intra-cluster correlations (ICC) ranging from 0.03 to 0.08 and a two-sided alpha of 0.05. These calculations assume an average sample size per clinic of 30 with a cluster sample size coefficient of variation (CV) of 25%. These numbers are based on estimated numbers of eligible patients seen monthly, obtained from a survey of the participating clinics. However, since these data will be obtained from the EHR, all eligible patients at each site will be included in the final analysis. For the target sample size of 22 clinics, there is at least 80% power to detect a difference of 15% if the ICC is 0.03 or lower. If the ICC is as high as 0.08, there is still at least 80% power to detect a difference of 20%. These calculations assume average utilization across all sites of 50%. However, power increases as rates move away from 50% in either direction.

**Table 2:** Total number of clinics needed to detect the specified difference in Reach as a function of the power, ICC, and difference

	80% power						90% power					
Diff/ICC	.03	.04	.05	.06	.07	.08	.03	.04	.05	.06	.07	.08
.15	22	26	29	33	36	40	30	34	39	44	48	53
.20	12	14	16	18	20	22	16	19	22	24	27	29

#### Effectiveness outcome

A key secondary objective is to assess the effect of mPATH™-CRC on screening rates (effectiveness), comparing 8 months of data prior to implementation to 8 months of data following implementation. The detectable difference (difference in screening rates pre vs post) depends on the sample size, power, ICC, and cluster sample size CV. We assume a screening rate in the pre-implementation group of 15%, as observed in the control group in the previous randomized-controlled efficacy study [43]. Table 3 shows the differences in screening rates that can be detected for the target sample size of 22 clinics with 80% and 90% power, for ICCs ranging from 0.03 to 0.06. Calculations assume an average monthly sample size of 30 patients per clinic in the EHR, a cluster sample size CV of 25%, a two-sided alpha of 0.05, and no change in the screening trends over time. All eligible patients at each site will be included in the final analysis as discussed for the primary outcome. With 22 clinics, a 10% difference in screening rates (15% vs 25%) can be detected with 80% power when the ICC is 0.04.

**Table 3:** Detectable difference in screening rates pre vs. post as a function of the power and the ICC (22 clinics)

	80% power				90% power			
ICC	.03	.04	.05	.06	.03	.04	.05	.06
	.087	.100	.113	.125	.102	.118	.133	.146

To evaluate whether the effect of mPATH™-CRC on screening rates differs by implementation strategy, the period (pre vs post) by strategy (“high touch” vs “low touch”) interaction can be evaluated. Based on the control group from the previous randomized-controlled efficacy study, we anticipate a screening rate of 15% for “low touch” and pre- implementation. An increase in the screening rate from 15% to 25% from pre to post, corresponds to an odds ratio (OR) of 3. Similarly, assuming a screening rate in the “low touch” group of 15%, and a screening rate of 25% in the “high touch” group, also corresponds to an OR of 3. By design, we anticipate half of the patients being in the “high touch” group and half in the post period, and that strategy and period are independent. Based on these assumptions, Table 4 shows the interaction OR that can be detected with 80% power for ICCs ranging from 0.03 to 0.06, assuming an average monthly sample size of 30 patients per clinic in the EHR (for 8 months pre and 8 months post), a cluster sample size CV of 25%, a two-sided alpha of 0.05, and no change in the screening trends over time.

**Table 4:** Detectable interaction odds ratio as a function of the ICC (22 clinics)

	80% power			
ICC	.03	.04	.05	.06
	2.903	3.462	4.097	4.819

### Recruitment {15}

The target sample size for this trial is 22 clinics. This study was presented to all primary care practices affiliated with Atrium Health Wake Forest Baptist. Clinic recruitment strategies included emails/phone calls to clinic personnel (practice managers and/or providers) and in-person pitch presentations. All clinic staff involved with the implementation of mPATH™ in study clinics are asked to participate in clinic staff surveys. Each participant receives a $10 gift card for each survey completed. Some clinic staff may also be asked to participate in semi-structured interviews. All interview participants receive a $50 gift card. There is no recruitment of individual patients in this study. Patient data is collected pragmatically through the EHR.

## Assignment of interventions: allocation

### Sequence generation {16a}

Randomization is stratified by clinic size, where small is defined as having 1–3 providers and large is defined as having 4 or more providers. Within each stratum, clinics are randomized in pairs (1 to the “high touch” strategy and 1 to the “low touch” strategy) using computer-generated random numbers.

### Concealment mechanism {16b}

Clinics are randomized by the study statistician at the time a pair of clinics is identified as ready for implementation of mPATH™.

### Implementation {16c}

The project manager notifies the study statistician when a pair of clinics within a given stratum are ready for randomization. The study statistician performs the randomization and notifies the project manager so appropriate training can be scheduled based on which clinic will be “high touch” and which will be “low touch.” Implementation of mPATH™ then occurs within the same month for a given pair of clinics.

## Assignment of interventions: blinding

### Who will be blinded {17a}

Clinic staff know that two implementation strategies are being evaluated, but they do not know the details of the two strategies and therefore do not know which strategy is being used at their clinic. Study staff are not blinded as they are necessary in implementing the two strategies.

### Procedure for unblinding if needed {17b}

There are no procedures for unblinding as there is no safety concern in this trial. Clinics may stop using mPATH™ at any point during the study, but unblinding is not necessary even for early discontinuation.

## Data collection and management

### Plans for assessment and collection of outcomes {18a}

Multiple data sources are used for the collection of outcomes and other trial data: (1) the mPATH™ database, (2) the EHR, (3) clinic personnel surveys, (4) semi-structured interviews, and (5) clinic contact and cost database.

#### mPATH™ database

The mPATH™ program database includes patients’ medical record numbers, age, sex, date/time of use, length of time in program, whether mPATH™-CheckIn and mPATH™-CRC modules are completed, and responses to questions. Questions asked within mPATH™ include screening questions (related to falls, safety, and depression), risk factor questions (personal history of CRC or colorectal polyps, family history of CRC, or visible blood in stool), and questions related to “self-order” of CRC screening tests. If patients are deemed as due for CRC screening, they are also asked to confirm if they have had a recent screening that may not have been recorded in the EHR. The front desk staff member who checks the patient in, the nursing staff member who rooms the patient, and the provider are also captured.

#### Electronic health record

For each study clinic, the EHR and appointment schedules will be queried to identify patients who completed visits, patient demographics, and patient clinical care data. Patient demographics include sex, race, ethnicity, and insurance status. In addition, patient zip code (to determine rural or urban residency) and geographic identifiers (GEOID; to determine socioeconomic status using the Area Deprivation Index [ADI] [70]) will be collected. Clinical care data include previous screening information to determine patient eligibility for CRC screening, and for those due for screening, whether the patient had a CRC screening test within 16 weeks of their visit to the clinic.

#### Clinic personnel surveys

Clinic personnel surveys are administered pre-implementation, and 6- and 12-month post-implementation using Research Electronic Data Capture (REDCap). If a clinic site decides to stop using the mPATH™ program prior to a scheduled follow-up survey, one final follow-up survey will be administered within 6 weeks of the practice discontinuing the use of mPATH™. All clinic personnel (e.g., administrators, nurses, providers) who are involved with the implementation of the mPATH™ program receive surveys. The pre-implementation survey includes basic demographic questions. All surveys assess constructs of the Technology Acceptance Model [55] and the Dynamic Sustainability Framework [58]. In addition, surveys evaluate implementation (Acceptability, Appropriateness, and Feasibility) from the staff perspective and assess organizational characteristics and individual characteristics. Experience with the mPATH™ program, including barriers and facilitators, is evaluated on the post-implementation surveys. Specific validated scales include the Acceptability of Intervention Measure (AIM), Intervention Appropriateness Measure (IAM), Feasibility of Intervention Measure (FIM) [68], and the Organizational Readiness for Implementing Change (ORIC) [71].

#### Semi-structured interviews

Semi-structured interviews are conducted with four members of each selected clinic: the clinic champion, one clinician, one front desk team member, and one medical assistant/nursing team member. The clinic champion assists in the identification of the other team members for interviews based on their experience with the mPATH™ program. An interview guide is used to explore how mPATH™-CRC was incorporated in the clinic’s work flow and factors that affected maintenance such as intervention adaptations, organizational characteristics, and the champion’s role. There is a separate version of the interview guide for clinics that have very low (or no) usage of mPATH™ during their participation in the study. Members of clinics that opt to discontinue the use of mPATH™ are interviewed as soon as practical after they discontinue use. Interviews will continue until thematic saturation is reached. These semi-structured interviews, expected to last approximately 30 min, are conducted by phone or video conference and analyzed by staff trained in qualitative methods. Interviews are audio recorded and transcribed verbatim. Transcripts will be imported into ATLAS.ti software [72], iteratively reviewed, coded by concept, and analyzed thematically to describe the implementation and sustainability experience. Themes will be compared among clinics to determine any differences.

#### Clinic contact and cost database

All study team contact with the clinics, and all technology costs associated with implementing and maintaining mPATH™ are recorded in a REDCap database. Data include date of contact, mode of contact (i.e., in-person, phone, email), person initiating contact, purpose of contact, and the roles of staff involved in the contact. These data will allow us to compare the cost of the “high touch” and “low touch” intervention strategies and associations between the amount of support given and specific implementation outcomes.

### Plans to promote participant retention and complete follow-up {18b}

Since patient data are collected by the mPATH™ program and by retrospective electronic chart review, there is no loss to follow-up in this study for patients. Clinic staff and providers participate in surveys and potentially interviews. To promote participation and retention, each participant receives a $10 gift card for each survey completed and all interview participants receive a $50 gift card. Reminders to fill out surveys are also sent to participants via REDCap, and if the completion rate is low, the study staff may go to the clinic in person to facilitate survey completion.

### Data management {19}

Patients use iPads to interact with the mPATH™ program. All iPad interaction data is temporarily stored on each iPad using secure Advanced Encryption Standard 256-bit encryption (AES-256) software to protect personal health information. The iPad software automatically sends the data to the central data storage server and removes the data from the iPad after confirmation that the data were sent and received. All study site databases are stored in an encrypted, Health Insurance Portability and Accountability Act (HIPAA)-compliant server with continuous data backup. EHR data will be queried by an honest broker and stored on an encrypted, HIPAA-compliant server with continuous data backup. Surveys are delivered to participants using REDCap, a web-based application for building and managing online surveys and databases. The clinic contacts and cost database is also maintained within REDCap. REDCap provides an intuitive interface for users to enter data, has real-time validation rules at the time of entry, maintains audit trails, and is HIPAA-compliant.

### Confidentiality {27}

Confidentiality will be protected by collecting only information needed to assess study outcomes, minimizing to the fullest extent possible the collection of any information that could directly identify patients, and maintaining all study information in a secure manner. To help ensure subject privacy and confidentiality, only a unique study identifier will appear in study datasets. An honest broker will mediate the electronic health record data queries to limit the exposure of patient identifying information. Planned analyses require full dates of visits (to determine the success of implementation over time), age, and 5-digit zip codes and GEOID (to determine rural or urban residency and ADI). Therefore, the honest broker will create a limited data set for analysis. The day that each clinic launches mPATH™ will be called day 0, with each subsequent or previous date recorded as an integer relative to day 0. This will add additional protections for patients by obscuring dates in the dataset. Any collected patient-identifying information corresponding to the unique study identifier will be maintained in a linkage file, stored separately from the data. The linkage file will be kept secure, with access limited to designated study personnel. Subject identifying information will be destroyed 3 years after the closure of the study, consistent with data validation and study design, producing an anonymous analytical data set. Data access will be limited to study staff. Data and records will be kept locked and secured, with any computer data password protected. No reference to any individual participant will appear in reports, presentations, or publications that may arise from the study.

### Plans for collection, laboratory evaluation, and storage of biological specimens for genetic or molecular analysis in this trial/future use {33}

See {26b} above; there will be no biological specimens collected.

## Statistical methods

### Statistical methods for primary and secondary outcomes {20a}

The primary objective of this study is to compare mPATH™-CRC Reach (proportion of participants who complete the mPATH™-CRC program or have risk factors identified by mPATH™-CheckIn) in the 6th month following implementation between the “high touch” and “low touch” strategies. This objective will be evaluated using a mixed effects logistic regression model with mPATH™-CRC Reach (yes vs. no) as the patient-level outcome, implementation approach (“high touch” vs. “low touch”) as the primary independent variable, site as a random effect, and the stratification factor clinic size (small vs. large) as a covariate per the design. A descriptive approach will also be utilized where mPATH™-CRC Reach is estimated and reported along with an exact 95% confidence interval for each month (for months 1–6 following implementation), and within socioeconomic strata (defined by the ADI and insurance status) for “high touch” and “low touch” clinics separately. This same basic approach will be used to compare other binary implementation outcomes (mPATH™-CRC Implementation Fidelity, mPATH™-CRC Maintenance, mPATH™-CheckIn Reach, and mPATH™-CheckIn Maintenance) between the “high touch” and “low touch” strategies. Continuous implementation outcomes (mPATH™-CRC Adoption, mPATH™-CRC Acceptability, mPATH™-CheckIn Adoption, mPATH™-CheckIn Acceptability) are measured at the provider/staff level, rather than the patient level. Linear mixed effects models will be used to compare these outcomes between the “high touch” and “low touch” strategies, with the implementation approach (“high touch” vs. “low touch”) as the primary independent variable, site as a random effect, and the stratification factor clinic size (small vs. large) as a covariate per the design.

The key secondary outcome of the effectiveness of mPATH™-CRC will be evaluated by comparing the proportion who complete CRC screening within 16 weeks of their visit to the clinic between a pre-implementation cohort (months 12–4 before implementation) and a post-implementation cohort (months 1–8 after implementation). Similar to the model described for mPATH™-CRC Reach above, the effectiveness of mPATH™-CRC will be evaluated using a mixed effects logistic regression model. For an overall assessment of the effectiveness, completing CRC screening within 16 weeks (yes vs. no) will be the patient-level outcome, the implementation cohort (pre vs. post) will be the primary independent variable, the site will be a random effect, and the stratification factor clinic size (small vs. large) will be a covariate per the design. Pre- vs post-implementation changes will also be compared between the “high touch” and “low touch” implementation strategies and between dose levels defined based on mPATH™-CRC usage by incorporating interactions into the mixed effects logistic regression models. In addition to the mixed model approach, an interrupted time series analysis (segmented regression) [73, 74] will also be used to estimate the effect of the intervention within each clinic (using interaction terms with clinic within a linear model) adjusting for any trend that was occurring in screening prior to the implementation. This model provides estimates of the time trends before and after the intervention as well as the change in screening rates due to the intervention. Linear contrasts will be used to compare the changes in screening rates and trends between the “high touch” clinics and the “low touch” clinics. The same overall approach will be followed for the outcome of having a CRC screening test ordered.

### Interim analyses {21b}

There are no planned interim analyses.

### Methods for additional analyses (e.g., subgroup analyses) {20b}

In secondary analyses, the effects of clinic-level covariates (e.g., volume, rurality, organizational culture) and patient-level covariates (e.g., age, sex, race/ethnicity) on implementation and effectiveness outcomes will be assessed. Implementation approach-by-covariate interactions will be included in the mixed models described above to determine if the effect of the implementation strategy differs depending on levels of the covariates. Using mixed models allows us to incorporate participant- and cluster-level covariates, but there are other analysis methods that could be employed. For example, a clinic-level analysis (e.g., analysis of covariance) on the mean implementation rates within each clinic, or another individual-level approach (e.g., generalized estimating equations) could be used. Alternative methods and their strengths and weaknesses are well-documented [75–77]. These alternate approaches may be used in sensitivity analyses to determine the effects of the modeling assumptions on the results.

### Methods in analysis to handle protocol non-adherence and any statistical methods to handle missing data {20c}

Since patient data are collected by the mPATH™ program and by retrospective electronic chart review, there is no loss to follow-up in this study for patients, and therefore missing patient-level data is not expected. Every effort will be made to get clinic personnel to fill out surveys, but some missing data are expected. All available survey data will be included without imputation.

### Plans to give access to the full protocol, participant level-data and statistical code {31c}

The full protocol and de-identified individual participant data that underlie the published or presented results will be shared with researchers who provide a methodologically sound proposal and have appropriate Institutional Review Board (IRB) approval.

## Oversight and monitoring

### Composition of the coordinating center and trial steering committee {5d}

The study team (including the PI, Co-Investigators, and project management staff) meets at least two times per month to discuss the study progress and review QA data. In addition, the study team formed an intervention change governance committee comprised of clinicians, information technology (IT) specialists, and program developers. This committee meets monthly to evaluate any requested changes to the mPATH™ program and their potential downstream effects. This committee has the final authority to determine whether a proposed change is developed, modified, or rejected.

### Composition of the data monitoring committee, its role and reporting structure {21a}

As this is a pragmatic study with a retrospective collection of clinical data, the only anticipated risks specific to this study are loss of data confidentiality. The mPATH™ study database is hosted on an encrypted secure server maintained by Wake Forest Information and Analytics Services (IAS). The database is password-protected, and only authorized individuals are granted access. IAS routinely monitors for any security weaknesses or breaches. In the unlikely event of a data breach, IAS will notify the PI who will convene an internal Data and Safety Monitoring committee to review the event and determine the appropriate response. The Data and Safety Monitoring committee will be comprised of the PI, Project Manager, study statistician, and study programmer. Other individuals may be added to the Data and Safety Monitoring committee at the PI’s discretion. In addition, the PI will promptly review any participant or other-reported concerns regarding the study. The PI will report any loss of data confidentiality or other adverse events to the Wake Forest University Health Sciences IRB and the NIH within 2 business days of the discovery of the event.

### Adverse event reporting and harms {22}

The only anticipated risks specific to this study are loss of data confidentiality. However, any unanticipated problems, serious and unexpected adverse events, deviations, or protocol changes will be promptly reported by the PI or a designated member of the research team to the Wake Forest University Health Sciences IRB and NIH if appropriate.

### Frequency and plans for auditing trial conduct {23}

Because this pragmatic trial is being conducted within the context of routine care delivery in primary care settings under a waiver of informed consent, no formal auditing procedures are in place. However, feedback about trial conduct will be received through clinic personnel surveys and interviews, and through phone calls with clinic champions for those study clinics randomized to the “high touch” strategy.

### Plans for communicating important protocol amendments to relevant parties (e.g. trial participants, ethical committees) {25}

All protocol amendments will be submitted to the Wake Forest University Health Sciences IRB and communicated to the study team and clinical teams as appropriate. The trial register at ClinicalTrials.gov will also be updated if relevant for the particular amendment.

### Dissemination plans {31a}

This study has the potential to meaningfully decrease morbidity and mortality by translating an evidence- based CRC-screening intervention into community practice, directly addressing the goals of Healthy People 2020 and the National Cancer Moonshot [44, 45]. However, this work will have a broader impact by identifying key factors to incorporate into implementation strategies to support the sustained use of similar technology-based primary care interventions, thus addressing an identified gap in the current literature [46–48]. We will share our results with the clinic sites and broadly disseminate our results through presentations at national meetings and publications. Wake Forest University Health Sciences has licensed mPATH™ to Digital Health Navigation Solutions, Inc. to make mPATH™ available to other institutions through commercialization.

## Discussion

The overall goal of this study is to evaluate the implementation and effectiveness of the mPATH™ program. While this overarching goal has not changed over time, many study details have been revised from the initial study conception. Changes were made based on formative work prior to the launch of the trial, and out of necessity because of the COVID-19 pandemic.mPATH-CRC™ was initially conceived as a single program that included both questions used to identify whether patients were due for CRC screening, as well as the CRC module (CRC screening decision aid and the ability to “self-order” a screening test) for patients who are due for screening. However, based on feedback provided by clinic staff through focus groups and semi-structured interviews [49], the original mPATH™-CRC program was adapted to meet check-in and workflow needs of the clinics. During this formative work, clinic staff reported that patients typically waited only 1–2 min in the waiting room, but then waited longer in the exam room. Therefore, we split the mPATH™ program into two distinct modules: mPATH™-CheckIn (used in the waiting room) and mPATH™-CRC (used in the exam room while waiting for the provider). mPATH™-CheckIn includes standard screening questions related to falls, safety, and depression that the participating health system requires nursing staff to ask all adult patients at each visit, which incentivizes clinics to use the program. This splitting of the program required a revision of the implementation outcomes, as mPATH™-CheckIn and mPATH™-CRC implementation now need to be evaluated separately; mPATH™-CheckIn outcomes are evaluated for all adult patients, whereas mPATH™-CRC outcomes only apply to patients eligible for CRC screening. Since the goal of the mPATH™ program is to improve CRC screening rates, the primary objective is related to the Reach of the mPATH™-CRC program, which is defined as completing the mPATH™-CRC module. Patients who have risk factors identified by mPATH™-CheckIn are also considered to be reached by the program because these patients are told to discuss screening with their provider.

The initial version of this study included a planned sample size of 28 clinics from both North Carolina (clinics affiliated with Atrium Health Wake Forest Baptist) and Kentucky (federally-qualified health centers). Implementation of mPATH™ in clinics was to start in North Carolina clinics first, with plans to expand to Kentucky later in the study. The two pilot clinics for this study were launched between August and October 2019, and the first clinic in the main trial was launched in November 2019. In March 2020, the trial had to be suspended because of the COVID-19 pandemic. At the time of suspension, 6 clinics had been randomized in the main trial (all in North Carolina); 4 clinics had been using mPATH™ for more than a month and 2 had been using mPATH™ for less than a week. The trial had to be suspended because elective procedures, including screening colonoscopies, were stopped at Atrium Health Wake Forest Baptist, which would impact the effectiveness outcomes for the trial. In addition, there were concerns around transmission of COVID-19 from using iPads in the clinics, patients not coming in to primary care clinics for routine clinical care, and added burden to clinic staff whose focus needed to be on patient care, not the trial. While the study team had initially hoped to resume the trial in Summer 2020 after elective procedures had restarted and some of the backlog was cleared, this was not possible given local spikes in COVID-19 and continuing concerns around implementation in primary care clinics.

By the beginning of 2021, there was general agreement between the study team and participating clinics that it was safe to resume the implementation of mPATH™ in primary care clinics. Vaccines were starting to be available, society had adapted to the pandemic, and other shared touchscreens were being used in outpatient settings. Based on this, a pilot clinic agreed to be re-launched in February 2021, and the main trial resumed in March 2021. With re-launch, mPATH™ iPad cleaning procedures were developed and approved by the Atrium Health Wake Forest Baptist Infection Control office and all mPATH™ iPads were enclosed in antimicrobial cases to ensure the safety of all involved. However, because the trial was suspended for a full year, there had been minimal data collection prior to the suspension, and there had been a lot of staff turnover at the study clinics, the study team decided that the best course of action was to start the study over at the time of re-launch (i.e., no data collected prior to re-launch will be used in final analyses for the trial). This included re-training the staff and collecting new baseline surveys. Clinics who were already in the study were not re-randomized (i.e., their assignment to either “high touch” or “low touch” stayed the same), but the time clock started over at the time of re-launch.

While the trial was able to be resumed in the North Carolina clinics, adding clinics in Kentucky was still a problem because of a travel restriction that was in place for Atrium Health Wake Forest Baptist employees. This meant that the study team with technical knowledge of the mPATH™ program would not the be able to travel to Kentucky for on-site implementation support. Given this logistical challenge, the study team made the difficult decision to restrict enrollment to only those clinics affiliated with Atrium Health Wake Forest Baptist. With the loss of both the Kentucky clinics and a year of time, the planed sample size was reduced from 28 clinics to 22 clinics, with approval from the NCI. Clinic recruitment was a challenge after re-launch, given the continuing pandemic and the reluctance of clinics to start something new during this difficult time. Despite this, 14 additional clinics were recruited and enrolled in the study after re-launch. Two of the original study clinics declined to participate at the time of re-launch (so will not contribute any data to the final analysis), leaving a final total sample size of 18 study clinics. Both clinics that declined to re-launch had been in the “low touch” arm. While this is a smaller sample size than intended, there is still at least 80% power to detect a difference of 20% in the primary outcome of Reach of mPATH™-CRC between implementation strategies even if the ICC is as high as 0.06. With 18 clinics there is also 82% power to detect a 10% difference in screening rates (15% vs 25%) pre- vs post-implementation, and an interaction OR of 3.3 can be detected to evaluate whether the effect of mPATH™-CRC on screening rates differs by implementation strategy when the ICC is 0.03. Therefore, despite the major challenges of conducting a trial during the COVID-19 pandemic, and all of the changes that are required, the current version of the study is still able to answer important questions around the implementation of the mPATH™ program and its effectiveness in improving screening rates for CRC. In addition, this work has the potential to have an even broader impact by identifying strategies to support the sustained use of other similar technology-based primary care interventions.

## Trial status

The current version of the protocol is dated 22 February 2023. The first pilot clinic was launched in August 2019, and the first clinic in the main trial was launched in November 2019. Because of the COVID-19 pandemic, all study activities were paused from March 2020-January 2021. A pilot clinic was re-launched in February 2021 and the first study clinic for the main trial was re-launched in March 2021. Patient recruitment will be complete at the end of March 2023. The protocol publication has been postponed until the end of the study because of the all changes required due to the COVID-19 pandemic and because study clinics are blinded to the details of the two implementation strategies.

## Data Availability

De-identified individual participant data that underlie the published or presented results will be shared with researchers who provide a methodologically sound proposal and have appropriate IRB approval. Use of the data will be limited to achieve the aims in their submitted proposal. To gain access, data requestors will need to sign a data access agreement.

## Abbreviations

ADI: Area Deprivation Index
AES-256: Advanced Encryption Standard 256-bit encryption
AIM: Acceptability of Intervention Measure
API: Application Programming Interface
CRC: Colorectal cancer
CT: Computed tomography
CV: Coefficient of variation
DNA: Deoxyribonucleic acid
EHR: Electronic health record
FIM: Feasibility of Intervention Measure
GEOID: Geographic identifier
HIPAA: Health Insurance Portability and Accountability Act
IAM: Intervention Appropriateness Measure
IAS: Information and Analytics Services
ICC: Intra-cluster correlation
IRB: Institutional Review Board
IT: Information technology
mHealth: Mobile health
mPATH™: Mobile PAtient Technology for Health
NCI: National Cancer Institute
NIH: National Institutes of Health
OR: Odds ratio
ORIC: Organizational Readiness for Implementing Change
PI: Principal Investigator
QA: Quality assurance
REDCap: Research Electronic Data Capture
TAM: Technology Acceptance Model
USPSTF: United States Preventive Services Task Force
